# Pangolin Distribution and Predicted Habitat Loss From the Nagmati Dam in Shivapuri Nagarjun National Park, Nepal

**DOI:** 10.1002/ece3.73441

**Published:** 2026-04-07

**Authors:** Pooja Lama, Asmit Subba, Kumar Paudel, Laxman Khanal

**Affiliations:** ^1^ Central Department of Zoology, Institute of Science and Technology Tribhuvan University Kathmandu Nepal; ^2^ Conservation Himalaya Kathmandu Nepal; ^3^ Greenhood Nepal Kathmandu Nepal; ^4^ IUCN SSC Pangolin Specialist Group London UK

**Keywords:** environmental compliance, habitat loss, infrastructure development, pangolins, threatened wildlife

## Abstract

Pangolins (genus *Manis*) are globally threatened mammals facing intense pressure from poaching, illegal trade, and habitat loss, particularly in human‐dominated landscapes. As habitat specialists, it is crucial to map site‐specific distribution and ensure human activities are not detrimental to their local population. This study examined pangolin distribution in the eastern part of Shivapuri Nagarjun National Park and assessed the potential impact of the proposed Nagmati Dam. We conducted continuous belt transect surveys (600 m long and 50 m wide) along the centroid of each grid cell across 101 grids (600 × 600 m each) at elevations of 1606–2422 m, recording 67 burrows (49 inactive, 18 active), indicating a clumped distribution. Moderate vegetation cover (using NDVI as a proxy) and slope were the primary environmental factors positively associated with the distribution of active pangolin burrows in Shivapuri Forest, with a substantial proportion of variation (McFadden's pseudo‐*R* ^2^ = 0.61). The proposed Nagmati Dam and its reservoir will inundate 25 ha of prime pangolin habitat and an additional 65.304 ha that likely fall within the home ranges and foraging areas of pangolins, resulting in substantial habitat loss. The Environmental Impact Assessment for the dam project overlooks this threat, failing to recognize its potential impact on pangolins or to propose mitigation measures to safeguard them during construction. We recommend robust conservation measures and a thorough reassessment of the dam's environmental impacts on this Critically Endangered species.

## Graphical abstract

Chinese pangolin burrows in Shivapuri Nagarjun National Park showed a clumped distribution influenced by slope and NDVI. The proposed Nagmati Dam and reservoir will inundate key pangolin habitats, causing significant habitat loss. The environmental impact assessment of the dam ignored these threats, highlighting the need for urgent reassessment to safeguard this critically endangered species.

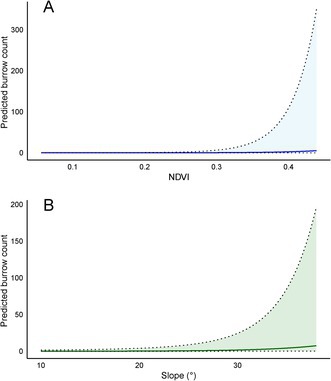

## 1 Introduction

The pangolin population has declined significantly throughout its range in recent decades due to poaching for bushmeat and the international trade of its scales for traditional medicines (Challender et al. 2019; Heinrich et al. 2017; Newton et al. 2008; Zhang et al. 2017). In addition to poaching, they face increasing habitat loss linked to rapid urbanization, agricultural expansion, deforestation, and infrastructure development (Challender et al. 2019; Heighton and Gaubert 2021). Multiple global initiatives aim to protect pangolin habitat and curb illegal trade, yet their effectiveness remains limited.

Pangolins have highly specific habitat requirements and relatively small home range (Shrestha, Bhattarai, et al. 2021a; Suwal et al. 2020a, 2020b; Waseem et al. 2020a, 2020b). Several environmental factors strongly influence their distribution and abundance, including the availability of termites and other prey (Hemachandra et al. 2014; Katuwal et al. 2017), leaf litter cover (Karawita et al. 2018), canopy cover (Bhandari and Chalise 2014; Wu et al. 2003), slope (Karawita et al. 2018; Shrestha, Bhattarai, et al. 2021a; Wu et al. 2004), proximity to water sources (Dhami et al. 2023; Panta et al. 2023), vegetation diversity and structure (Odewumi and Ogunsina 2018; Sharma, Rimal, Zhang, et al. 2020), and the intensity of human activities (Dhami et al. 2023; KC et al. 2024; Subba et al. 2024). Infrastructure development, such as road construction, urban expansion, dams, and hydropower projects, leads to habitat fragmentation, loss of foraging grounds, and elevated poaching risk due to increased access near pangolin habitat (Khatiwada et al. 2022; Sharma, Rimal, Zhang, et al. 2020; Subba et al. 2024). At the same time, growing evidence reveals the complexity of pangolin conservation, with new species and distinct genetic lineages continuing to be described (Gu et al. 2023; Hogan et al. 2025; Koju et al. 2025; Wangmo et al. 2025).

In Nepal, two recognized pangolin species, Chinese pangolin ( *Manis pentadactyla* ) and Indian pangolin ( *M. crassicaudata* ), have been reported from many parts of the country (Jnawali et al. 2011; DNPWC 2019; DNPWC and DoF 2018). The Chinese pangolin has been recorded in over 25 districts and 9 protected areas, including Shivapuri Nagarjun National Park (SNNP) (DNPWC and DoF 2018; Khatiwada et al. 2022; Suwal et al. 2020a, 2020b; Thapa et al. 2014). Shivapuri Nagarjun National Park is recognized as a key habitat for the pangolins (DNPWC 2019). The park comprises two forest blocks, Shivapuri Forest and Nagarjun Forest, which were once part of a continuous landscape but are now separated by human activities (Poudyal et al. 2023). Although several studies have documented pangolins in the western forest patch of Shivapuri‐Nagarjun National Park (Aryal and Poudel 2018; Bhandari and Chalise 2014; Dhital et al. 2020), the eastern forest block, Shivapuri Forest, remains uncovered. This gap is particularly concerning given rapid landscape change from new roads, settlements, and dam construction in the eastern block of the park (BIP 2026). Understanding pangolin distribution and habitat requirements in this area is therefore essential so that such critical habitats can be protected.

Dam construction within natural pangolin habitats is likely to have far‐reaching ecological consequences that extend beyond pangolins to other wildlife if potential impacts are not properly assessed and mitigated. The Nagmati Dam in SNNP is a 94.5 m high dam (capacity: 8.5 million m^3^), under financial support of the Asian Development Bank, being constructed in known pangolin habitat, yet its planning has not adequately considered potential impacts or mitigation measures (BIP 2026). The Environmental Impact Assessment (EIA) for the Nagmati Dam project did not sufficiently evaluate adverse impacts on globally threatened species, including the Critically Endangered Chinese pangolin and four Vulnerable mammals: sambar ( *Rusa unicolor* ), clouded leopard ( *Neofelis nebulosa* ), Asiatic black bear ( *Ursus thibetanus* ), and mainland serow ( *Capricornis sumatraensis* ). Moreover, the EIA includes no species‐specific conservation strategies to reduce the effects of dam construction. Accordingly, this study aimed to: (1) map the distribution of pangolins in the eastern part of SNNP; (2) identify key habitat requirements of pangolins in this area; (3) examine the potential loss of critical pangolin habitats due to the Nagmati Dam; and (4) provide actionable recommendations to mitigate these impacts and protect pangolins in SNNP. In this context, we formulated three central hypotheses. First, we hypothesized that pangolin distribution in Shivapuri Forest is non‐random and influenced by specific habitat characteristics, predicting that burrow occurrence would be significantly associated with moderate canopy cover, particular slope ranges, and higher vegetation index values, consistent with patterns in the adjacent Nagarjun Forest. Second, we hypothesized that proximity to human infrastructure negatively affects pangolin habitat use, predicting that burrow density would decline with decreasing distance to roads, trails, and the dam construction site. Third, we hypothesized that construction of the Nagmati Dam will directly and indirectly degrade high‐quality pangolin habitat in the park, predicting that spatial overlays of its distribution with the project footprint would show substantial loss of high‐suitability habitat to construction activities.

The situation of infrastructure development in SNNP is representative of broader development trends in Nepal. The country is advancing numerous hydropower and dam projects, several of which overlap with pangolin habitats (DHI Group 2013a, 2013b; World Bank 2012).

## 2 Methods

### 2.1 Study Area

**FIGURE 1:**
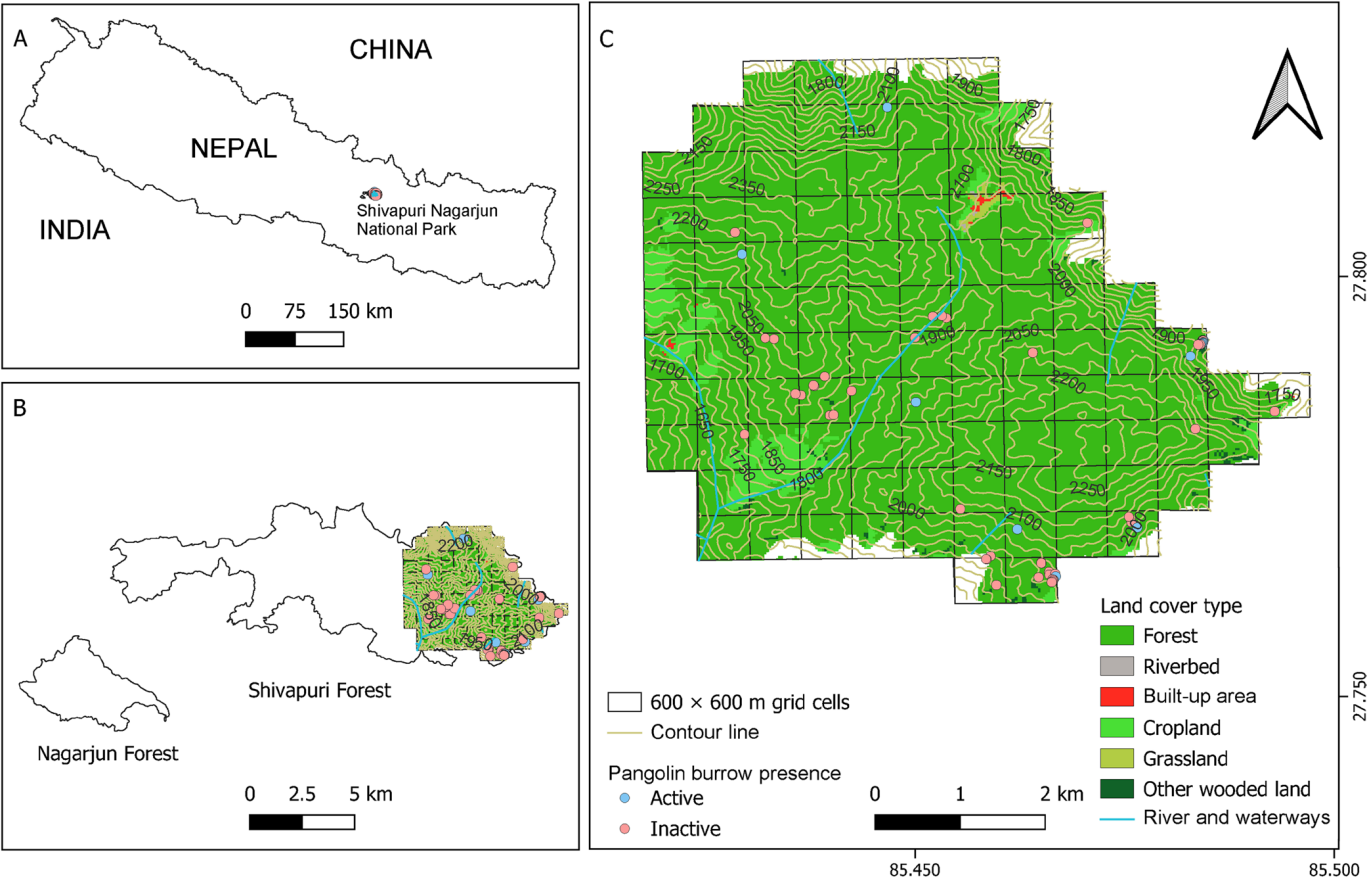
Map of the study area with elevation range shown in contour line and survey grid cells; (A) Map of Nepal showing Shivapuri Nagarjun National Park; (B) Map showing the two forest patches of the park and survey areas; and (C) Map of the study area showing survey grid cells and Chinese pangolin burrow records.

This study was conducted in Shivapuri Forest, located in the eastern sector of Shivapuri Nagarjun National Park (SNNP), central Nepal (Figure 1), with research approval from the Department of National Parks and Wildlife Conservation, Government of Nepal. The park encompasses rugged and heterogeneous terrain characterized by valleys, ridges, gentle to steep slopes, and small mountains, with elevations ranging from 960 to 2732 m above sea level (a.s.l.) at Shivapuri Peak. The study area includes forested zones and inhabited settlements such as Mulkharka and parts of Chilaune and Okhreni, which fall under the Sundarijal–Shivapuri Forest User Committee. Despite its ecological significance, SNNP faces major conservation threats, including tourism pressure, habitat destruction, human encroachment, illegal wildlife trade, agricultural pesticide use, and limited public awareness regarding conservation (Koju 2018).

### 2.2 Pangolin Burrow Survey

The study area was divided into 600 × 600 m grid cells following Chinese pangolin monitoring guidelines (DNPWC 2019) to assess burrow distribution across habitat conditions. Of the 105 grid cells established, 101 were surveyed between September and December 2023; four grid cells were excluded because steep terrain and lack of trails made them inaccessible. Given the species' nocturnal, elusive, and fossorial behavior (Willcox et al. 2019), we used burrows as indicators of pangolin presence (Wilson and Delahay 2001). Burrows were classified into two categories based on physical characteristics: active burrows, showing signs of recent use such as fresh digging, moist soil, or footprints; and inactive burrows, showing evidence of past excavation but no recent activity, indicated by hardened soil or vegetation overgrowth (DNPWC 2019).

To improve detectability, we established a continuous belt transect 600 m long and 50 m wide along the centroid of each grid cell, following a standardized protocol designed to maximize detection probability across contrasting habitat structures (DNPWC 2019; Subba et al. 2024). Environmental data were collected every 100 m along each transect (Mackenzie and Royle 2005), yielding six sampling points per grid cell. Values from these six points were averaged to derive grid cell level habitat conditions (Subba et al. 2024). All surveys were conducted systematically by trained observers between 08:00 and 16:00 h, although we acknowledge that detectability likely varied with terrain and vegetation density.

Vegetation was sampled systematically using six quadrats (10 m × 10 m) per transect, placed at 100 m intervals (DNPWC 2019). For trees within quadrats, we measured circumference at breast height (CBH) > 31.4 cm and converted it to diameter at breast height (DBH). Species‐specific counts and CBH values were recorded. We calculated the Important Value Index (IVI) for trees as IVI = Relative Frequency + Relative Density + Relative Dominance (Adam et al. 2007; Maingi and Marsh 2006), where:

$$\text{Relativefrequency}=\frac{\text{Number of plots that contain}\;a\;\text{species}}{\text{Number of}\;\mathrm{all}\;\text{plots}}\times 100$$

$$\text{Relativedensity}=\frac{\text{Number of individuals of}\;a\;\text{species in}\;\mathrm{all}\;\text{plots}}{\text{Total number of individuals of}\;\mathrm{all}\;\text{species in}\;\mathrm{all}\;\text{plots}}\times 100$$

$$\text{Relativedominance}=\frac{\text{Total basal area of}\;a\;\text{species in}\;\mathrm{all}\;\text{plots}}{\text{Total basal area of}\;\mathrm{all}\;\text{species in}\;\mathrm{all}\;\text{plots}}\times 100$$

Road, settlement, and water‐body layers were obtained as shapefiles from OpenStreetMap (OSM 2021) and imported into QGIS version 3.28 (QGIS Development Team 2022), where the NNJoin tool was used to calculate Euclidean distances from each grid cell to the nearest road, settlement, and water body. Landsat‐8 imagery from October 2023 (https://earthexplorer.usgs.gov/) was processed in QGIS 3.28, using Band 4 (red) and Band 5 (near‐infrared) in the Raster Calculator to compute the Normalized Difference Vegetation Index (NDVI = [Band 5—Band 4] / [Band 5 + Band 4]; Kshetri 2018; Malik et al. 2019) at centroids of each grid cell.

A 12.5 m resolution digital elevation model (DEM) was obtained from the Alaska Satellite Facility (ASF 2021) and used in QGIS to derive mean slope values for each quadrat with the Slope tool. Aspect values at the centroid of each grid cell were extracted using the Aspect tool and the GRASS “aspect” module (GRASS Development Team, 2020). Soil color and soil type were recorded in situ during field surveys, and for each grid cell we assigned the dominant soil color and soil type observed across its six quadrats for subsequent analyses.

### 2.3 Spatial Context of Nagmati Dam Project

Spatial data for the proposed Nagmati Dam project were obtained from the Environmental Impact Assessment (EIA) report prepared by the Government of Nepal, Ministry of Energy, Water Resources and Irrigation, Department of Water Resources and Irrigation, Nagmati Dam Project, Lalitpur, headquartered at Gaurighat, Guheswori, Kathmandu. The project comprises a concrete gravity dam on the Nagmati River, along with the reservoir inundation area, access roads, construction camps, spoil disposal sites, and tunnel alignments within Shivapuri Nagarjun National Park, central Nepal. The dam axis location, auxiliary infrastructure footprints, and estimated reservoir extent (approximately 480,426 m^2^) were digitized and georeferenced in QGIS. These project components were overlaid with the 600 × 600 m pangolin burrow survey grid cells to quantify spatial overlap between proposed infrastructure and surveyed habitats. This spatial integration anchored the ecological analysis in verifiable project design parameters and enabled assessment of habitat loss and fragmentation specifically associated with the Nagmati Dam project.

### 2.4 Data Analyses

Continuous variables were standardized by subtracting their means and dividing by their standard deviations, following Subba et al. (2024). To assess the spatial distribution of pangolin burrows (active and inactive combined), we calculated the variance‐to‐mean ratio $\frac{S^{2}}{a}$, where *a* is the mean number of burrows (*x*) per transect and $S^{2}=\frac{1}{n}\sum {\left(x-a\right)}^{2}$; values of 1 indicate a random distribution, values < 1 indicate a uniform pattern, and values > 1 indicate a clumped pattern (Odum 1971). For subsequent ecological and threat analyses, only active burrows were included.

Before fitting the global model, we assessed all predictors for multicollinearity. An initial check of variance inflation factors (VIF) for the full model, which included soil color, showed low values for all predictors (VIF < 1.62; Table S1). However, Fisher's exact test revealed a significant association between the categorical variables soil type and soil color (*p* < 0.05). To avoid overparameterization and improve parameter stability, we retained only one of these variables. Based on a lower AIC in univariate models and stronger evidence of biological relevance to digging behavior, we retained soil type in the final analysis. A subsequent VIF analysis for the final model (excluding soil color) indicated no multicollinearity concerns (all VIF < 1.61; Table S2).

We first fitted a generalized linear model (GLM) with a Poisson error distribution to identify environmental predictors of burrow counts, using the total number of active feeding and resting burrows per grid cell as the response variable and nine predictors: elevation, slope, IVI, distance to settlement (DS), distance to road (DR), distance to water source (DWS), NDVI, aspect, and soil type. A Pearson χ^2^ dispersion ratio indicated mild overdispersion (≈1.84), but simulation‐based diagnostics using the *DHARMa* package (Hartig 2024) showed no significant overdispersion (*p* > 0.8; Figure S1) and no evidence of zero inflation (*p* > 0.72; Figure S2). We also assessed spatial autocorrelation in the model residuals using Moran's I. The results indicated no significant spatial structure, suggesting that spatial dependence did not influence the parameter estimates. For robustness, we also fitted a negative binomial GLM, which produced qualitatively similar results (Figures S3 and S4) but did not improve model fit (ΔAIC ≈2, likelihood ratio test *p* > 0.05). On the basis of parsimony (Burnham and Anderson 2002) and diagnostic support, we retained Poisson GLM for inference.

Model selection was performed using AICc, considering the model with the lowest AICc as the best‐supported (Burnham and Anderson 2002). Given the relatively low weight of the top‐ranked model, we used the *MuMIn* package (Bartoń 2023) to perform model averaging across candidate models with ΔAICc < 2. All analyses were conducted in R 4.3.3 (R Core Team 2020).

## 3 Results

### 3.1 Distribution of Pangolin and Its Habitat

We detected 67 pangolin burrows along 21 belt transects (20.79% of 101 grid cells). Most burrows were inactive (*n* = 49, 73.13%), whereas only 18 (26.87%) were active. The variance‐to‐mean ratio for burrow counts was 8.99, indicating a clumped distribution of burrows across the study area (Figure 1).

Burrows occurred at a mean elevation of 2076.54 ± 170.73 m and on slopes averaging 19.73° ± 5.87° (Table S3). The mean distance from burrow‐present grid cells to the nearest water source was 1393.28 ± 1022.06 m, while mean distances to the nearest settlement and road were 566.17 ± 384.90 m and 223.14 ± 261.00 m, respectively. The mean NDVI value of burrow‐present grid cells was 0.29 ± 0.09, and the mean IVI of trees in these grid cells was 116.22 ± 57.30.

Among tree species, the highest proportion of burrows was recorded near *Alnus nepalensis* (*n* = 18, 26.87%), followed by *Pinus roxburghii* (*n* = 15, 22.39%) and *Rhododendron arboreum* (*n* = 7, 10.45%) (Table 1). Burrows were most common at 1950–2150 m a.s.l. (*n* = 33), on slopes of 15°–30° (*n* = 32), on west‐facing aspects (*n* = 24), and in brown soils (*n* = 61), with a preference for sandy soil texture (*n* = 37). Most burrows were located within 0–700 m of settlements (*n* = 56) and 200–400 m of roads (*n* = 33), and burrow abundance peaked at 2100–2800 m from the nearest water body (*n* = 19; Table S3).

**TABLE 1:** Major tree species and associated pangolin burrow abundance in survey grid cells of Shivapuri Forest, Shivapuri Nagarjun National Park, Nepal.

Name of tree species	Frequency of trees	Relative frequency of trees	No. of burrows	Relative abundance of burrows
Nepal alder ( *Alnus nepalensis* )	2	0.02	18	0.27
Chir pine (Pinus roxburghii)	4	0.04	15	0.22
Rhododendron (Rhododendron arboreum)	8	0.08	7	0.1
Japanese blue oak ( *Quercus glauca* )	23	0.23	5	0.08
European nettle tree ( *Celtis australis* )	1	0.01	5	0.08
Nepal camellia (Camellia kissi)	1	0.01	5	0.08
Oval‐leaf yonia (Lyonia ovalifolia)	11	0.11	4	0.06
Indian chestnut (Castanopsis indica)	3	0.03	2	0.03
Nepali hog plum ( *Choerospondias axillaris* )	1	0.01	2	0.03
Sapphire berry ( *Symplocos paniculata* )	10	0.1	1	0.02

### 3.2 Factors Affecting the Distribution of Pangolins

**FIGURE 2:**
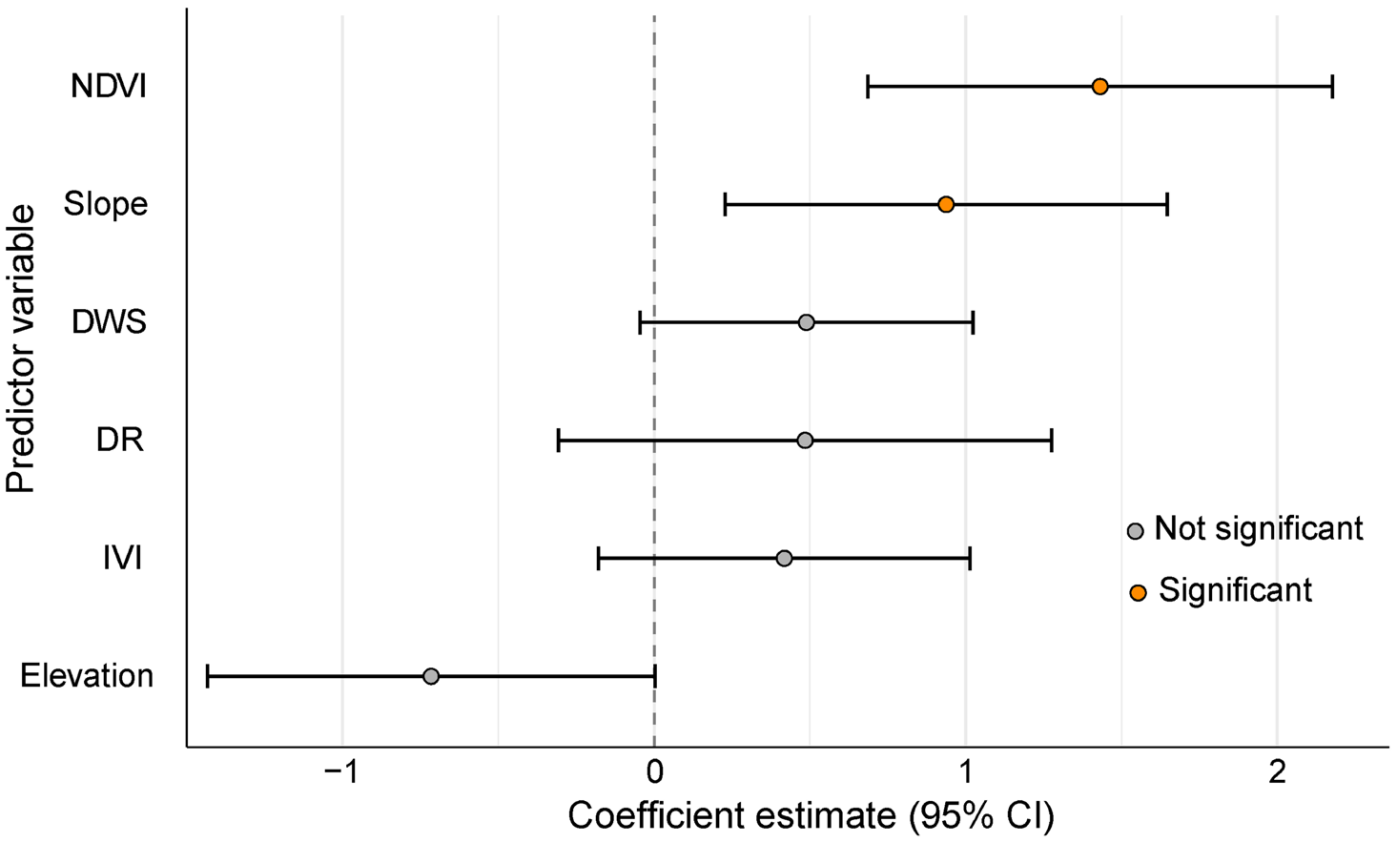
Forest plot of model‐averaged conditional coefficients (95% CI) for predictors of active pangolin burrow counts. Orange points indicate variables with *p* < 0.05; gray points indicate *p* ≥ 0.05. The dashed vertical line represents a coefficient of zero (no effect). Predictors are ordered by effect size. Model selection and averaging were performed using AICc (ΔAICc < 2).

**FIGURE 3:**
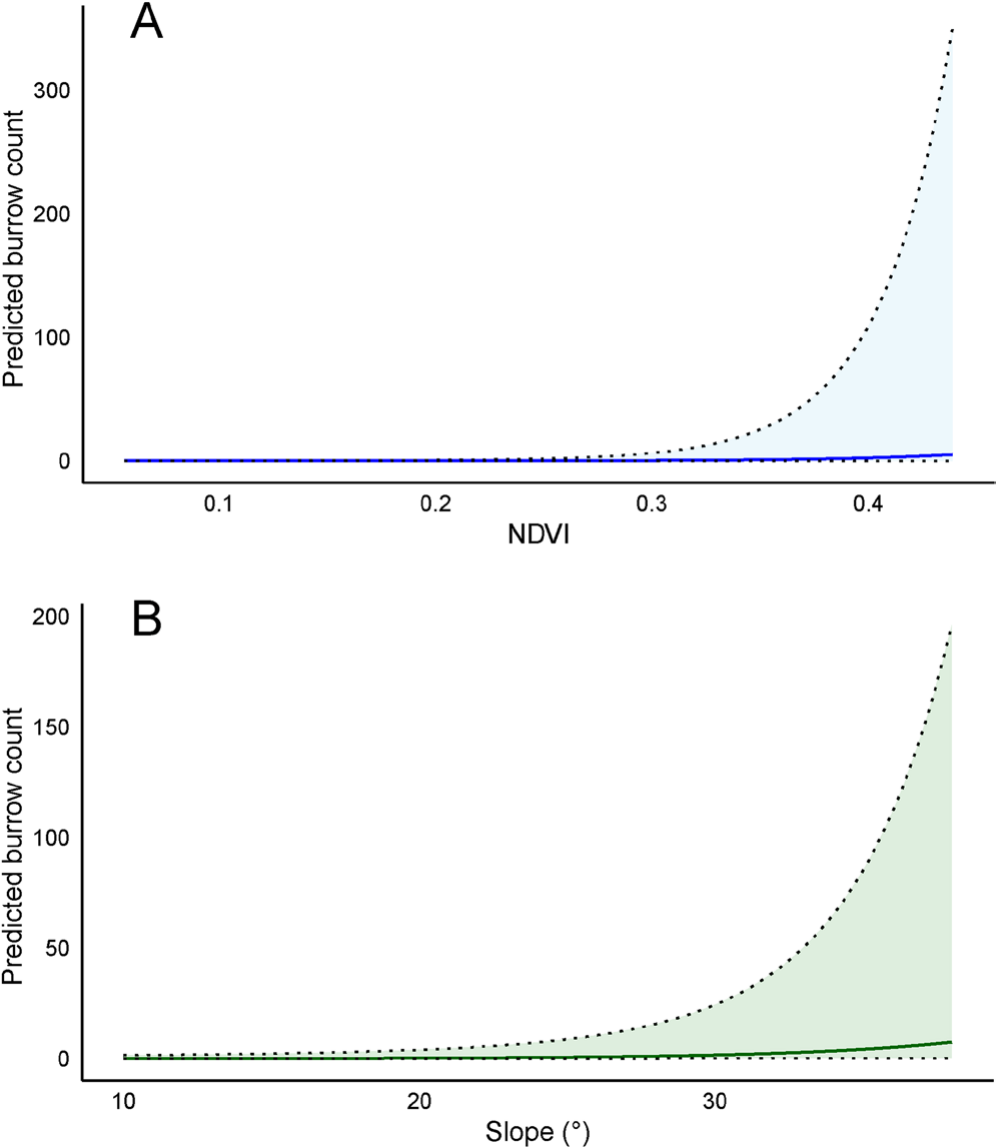
Partial effects of (A) slope and (B) normalized difference vegetation index (NDVI) on the predicted number of active pangolin burrows from the Poisson generalized linear model, with 95% confidence intervals.

The GLM with Poisson error distribution resulted in the top model with an AICc of 81.72 and an Akaike weight of 0.32 (Table 2). Model averaging of the top‐ranked models identified significant association of NDVI and slope with Chinese pangolin burrow abundance (Figure 2). NDVI values ranging from 0.06 to 0.44, representing areas with low to moderate vegetation density, showed a statistically significant positive association (Figure 3a) with the number of pangolin burrows (C.I. = 0.69–2.18, *p* < 0.05). Slope values between 10° and 38° also exhibited a significant positive association (Figure 3b) with burrow numbers (C.I. = 0.23–1.65, *p* < 0.05; Table S4).

**TABLE 2:** General linear models in AICc < 2 (top models) used for model averaging to describe the relationship between Pangolin burrow abundance and habitat characteristics.

S.N.	Variables	df	logLik	AICc	Delta	Weight
1	DWS + Elevation + NDVI + Slope	5	−35.54	81.72	0	0.32
2	DR + DWS + Elevation + NDVI + Slope	6	−34.87	82.63	0.91	0.21
3	DWS + Elevation + IVI + NDVI + Slope	6	−34.94	82.77	1.05	0.19
4	DWS + IVI + NDVI + Slope	5	−36.39	83.42	1.69	0.14
5	IVI + NDVI + Slope	4	−37.51	83.43	1.71	0.14

Abbreviations: AICc, Akaike information criterion corrected for small sample size; df, degrees of freedom; DR, distance to road; DWS, distance to water source; Elevation (m); IVI, Importance Value Index; logLik, log‐likelihood; NDVI, normalized difference vegetation index; slope (°).

### 3.3 Potential Implications of the Proposed Dam on Pangolins

**FIGURE 4:**
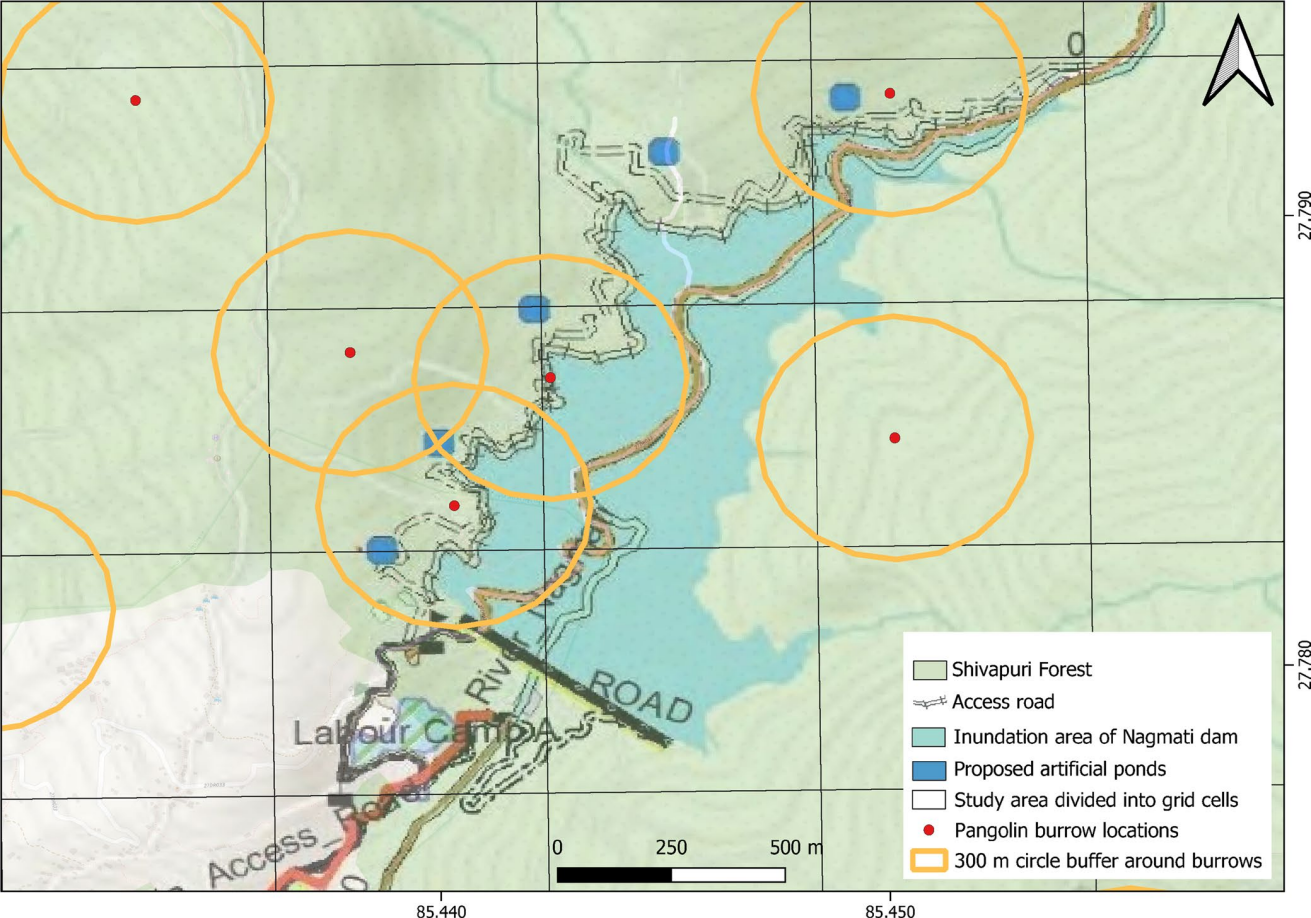
Spatial overlap between pangolin home‐range buffers (600 m radius around active burrows) and the proposed Nagmati Dam and reservoir footprint in Shivapuri Forest, Shivapuri Nagarjun National Park, showing areas of potential habitat inundation.

The proposed Nagmati Dam and reservoir spatially overlap with areas where pangolin burrows were recorded, suggesting that some habitats currently used by the species may fall within the projected dam footprint. To explore this spatial relationship, we generated a 600 m diameter buffer around 17 active burrows, excluding 50 burrows that were clustered within 400 m of each other to reduce spatial redundancy. Among these, buffers surrounding three burrows overlapped with approximately 251,642.49 m^2^ (0.25 km^2^) of the proposed dam and reservoir area (Figure 4). While this overlap indicated that portions of the area used by pangolins could potentially be affected by the proposed infrastructure, the burrow‐based buffer approach represents only a coarse proxy for home‐range extent and does not provide direct evidence of population‐level impacts. A more comprehensive assessment, potentially incorporating detailed movement data, spatial null models, or population viability analyses, would be necessary to robustly evaluate the extent to which the proposed infrastructure could influence pangolin habitat use or long‐term persistence in the area.

## 4 Discussion

### 4.1 Distribution of Pangolin Burrows in Shivapuri Nagarjun National Park

Pangolin burrows showed a clumped distribution in the eastern part of Shivapuri Nagarjun National Park, with NDVI and slope emerging as the two most important predictors. This clumped pattern is consistent with results from other forests in Nepal (Bhandari and Chalise 2014; Kunwar 2023; Lamichhane and Pokhrel 2019; Panta et al. 2023; Rai et al. 2019; Suwal 2011) and likely reflects strong habitat specificity, localized prey availability, and species‐specific behaviors (Dhami et al. 2023; Kunwar 2023; Suwal et al. 2020a, 2020b).

Burrows were recorded on slopes between 13° and 32° (Table S5), suggesting a preference for moderate gradients. Such slopes may facilitate digging, enhance mobility, and reduce the risk of rain‐induced soil erosion (Acharya et al. 2021; Dorji 2017; Tamang et al. 2022). Our GLM showed a positive effect of slopes on burrow distribution, possibly because these areas offer suitable digging conditions and higher prey densities (Sharma, Sharma, Chaulagain, et al. 2020; Sharma, Sharma, Katuwal, and Belant 2020). Excavating burrows on steeper slopes may also conserve energy (Heath and Vanderlip 1988; Wu et al. 2003) and reduce damage to burrow entrances from heavy rainfall (Hua et al. 2015). Although all burrows were classified during field surveys, only active burrows were used in spatial modeling.

The GLM revealed that NDVI values in the range of 0.2–0.38 (within the broader 0.06–0.44 range) were associated with higher burrow counts, indicating a preference for moderately vegetated habitats. NDVI has been identified as a key predictor of pangolin presence in several landscapes (Liu et al. 2024; Mouafo et al. 2023; Waseem et al. 2020a, 2020b). This preference may reflect higher termite availability in areas with less dense canopy cover (Hemachandra et al. 2014; Panta et al. 2023) and improved burrow ventilation (Hemachandra et al. 2014). Overall, our results highlight the pangolin's reliance on specific ecological conditions (Acharya et al. 2021; Sharma, Rimal, Zhang, et al. 2020; Thapa et al. 2014). The global model exhibited a strong fit (McFadden's pseudo‐*R* ^2^ = 0.61), indicating that the included environmental and habitat variables collectively explain a large proportion of the variation in burrow counts. However, the effect sizes (incidence rate ratios) for individual predictors were modest for some variables (e.g., DWS, DR) and were estimated with considerable uncertainty, as reflected by wide confidence intervals. This suggests that while the overall model is informative, unmeasured factors may also contribute to burrow site selection, and the precision of individual effect estimates warrants cautious interpretation. Because this study covers only a small portion of the species' range and does not explicitly account for imperfect detection, the habitat relationships identified here should be extrapolated cautiously to other landscapes until further validation is available.

### 4.2 Threats to Pangolins From the Nagmati Dam

The proposed 90 m high dam, with a storage capacity of up to 8 × 10^6^ m^3^, has the potential to affect pangolin habitat within the study area. Spatial overlay analysis indicated that approximately 25 ha of predicted suitable habitat lies within the direct impact zone, with an additional 65.304 ha potentially subject to indirect impacts associated with construction activities. These estimates are derived from modeled habitat suitability and should therefore be interpreted with caution. The GLM results identified NDVI and slope as main predictors of burrow occurrence. The mean NDVI (0.3426) and slope (17°) of grid cells projected to be inundated fall within ranges associated with higher burrow occurrence, suggesting that some areas of suitable habitat may be affected by reservoir formation.

Reservoir inundation may result in localized habitat loss, while construction‐related activities could contribute to habitat fragmentation and alterations in soil conditions, with potential implications for prey availability. However, the magnitude and spatial extent of such impacts remain uncertain given the limitations of the study, including reliance on indirect indicators of habitat suitability and the absence of temporal data. Although a detailed feasibility study and Environmental Impact Assessment (EIA) have been completed for this project (Bagmati Improvement Project 2025), impacts on pangolins are not explicitly considered.

At the local scale, this study provides an initial assessment of pangolin habitat associations in Shivapuri Forest within Shivapuri Nagarjun National Park and may inform future research and management interventions. More broadly, it contributes to the limited empirical understanding of pangolin habitat use in mid‐hill ecosystems of Nepal. Given the species' sensitivity to disturbance and cryptic behavior, further targeted investigations are warranted to better evaluate potential responses to infrastructure development. In this context, additional assessment focusing on species‐specific impacts could support the development of appropriate mitigation measures, such as maintaining habitat connectivity and minimizing disturbance during construction activities.

## 5 Conclusion

This study provides important insights into the distribution of pangolins in the eastern part of Shivapuri Nagarjun National Park. Moderate NDVI and slope emerged as the primary factors positively influencing the distribution of active pangolin burrows in Shivapuri Forest. The proposed Nagmati Dam project overlaps with habitat of the Critically Endangered Chinese pangolin, yet the existing Environmental Impact Assessment does not acknowledge this impact or propose mitigation measures to safeguard the species during dam construction. We emphasize the urgent need for a supplementary environmental assessment before construction proceeds. Given the lack of comprehensive data on pangolins in Shivapuri Forest and its buffer zone, we also recommend further research to inform appropriate management plans and conservation strategies.

## Author Contributions

**Pooja Lama:** conceptualization (equal), data curation (lead), formal analysis (equal), funding acquisition (lead), investigation (lead), methodology (equal), resources (equal), validation (equal), writing – original draft (lead). **Asmit Subba:** formal analysis (equal), methodology (equal), validation (equal), visualization (equal), writing – original draft (equal), writing – review and editing (equal). **Kumar Paudel:** conceptualization (equal), methodology (equal), resources (equal), supervision (equal), writing – review and editing (equal). **Laxman Khanal:** conceptualization (lead), project administration (equal), resources (lead), supervision (lead), validation (equal), writing – review and editing (equal).

## Funding

This work was supported by WWF Nepal and Greenhood Nepal, Kathmandu.

## Ethics Statement

Permission for the research was obtained from the Department of National Parks and Wildlife Conservation, Government of Nepal. During the study, no animals were handled.

## Conflicts of Interest

The authors declare no conflicts of interest.

## Supporting information


**Figure S1:** DHARMa nonparametric dispersion test based on residuals fitted versus simulated of Poisson error distribution.
**Figure S2:** DHARMa zero‐inflation test via comparison to expected zeros with simulation in Poisson error distribution.
**Figure S3:** DHARMa nonparametric dispersion test based on residuals fitted vs. simulated of negative binomial GLM.
**Figure S4:** DHARMa zero‐inflation test via comparison to expected zeros with simulation in negative binomial GLM.
**Table S1:** VIF values of full model.
**Table S2:** VIF values of full model except soil color.
**Table S3:** Summary statistics (mean ± standard deviation, SD) of environmental and habitat variables across all sampled grid cells (*n* = 101) and grid cells with pangolin burrows (*n* = 21) in Shivapuri Nagarjun National Park, Nepal.
**Table S4:** Poisson generalized linear model examining effects of environmental and habitat variables on active pangolin burrow counts across surveyed grid cells in Shivapuri Nagarjun National Park, Nepal.
**Table S5:** Chinese pangolin burrows recorded under different ecological conditions.

## Data Availability

The data used in this study is available from the following link of Science Data Bank (ScienceDB): https://doi.org/10.57760/sciencedb.28599.
