# Supplementary material for: Pangolin Distribution and Predicted Habitat Loss From the Nagmati Dam in Shivapuri Nagarjun National Park, Nepal
Source: Ecol Evol. 2026 Apr 7;16(4):e73441. doi: 10.1002/ece3.73441 (PMC13054232; doi:10.1002/ece3.73441)
Supplement: Supplementary file 1 — Figure S1: DHARMa nonparametric dispersion test based on residuals fitted versus simulated of Poisson error distribution. Figure S2: DHARMa zero‐inflation test via comparison to expected zeros with simulation in Poisson error distribution. Figure S3: DHARMa nonparametric dispersion test based on residuals fitted vs. simulated of negative binomial GLM. Figure S4: DHARMa zero‐inflation test via comparison to expected zeros with simulation in negative binomial GLM. Table S1: VIF values of full model. Table S2: VIF values of full model except soil color. Table S3: Summary statistics (mean ± standard deviation, SD) of environmental and habitat variables across all sampled grid cells (n = 101) and grid cells with pangolin burrows (n = 21) in Shivapuri Nagarjun National Park, Nepal. Table S4: Poisson generalized linear model examining effects of environmental and habitat variables on active pangolin burrow counts across surveyed grid cells in Shivapuri Nagarjun National Park, Nepal. Table S5: Chinese pangolin burrows recorded under different ecological conditions. [file ECE3-16-e73441-s001.docx]

**Supplementary File 1**

**Pangolin Distribution and Predicted Habitat Loss from the Nagmati Dam in Shivapuri Nagarjun National Park, Nepal**

Pooja Lama^1^, Asmit Subba^1,2^, Kumar Paudel^3,4^, Laxman Khanal^1^*

^1^ Central Department of Zoology, Institute of Science and Technology, Tribhuvan University, Kathmandu 44618, Nepal

^2^ Conservation Himalaya, Chandragiri-14, Kathmandu 44619, Nepal

^3^ Greenhood Nepal, Kathmandu, Nepal

^4^ IUCN SSC Pangolin Specialist Group

Corresponding author: Laxman Khanal; Email: [laxman.khanal@cdz.tu.edu.np](mailto:laxman.khanal@cdz.tu.edu.np)

Table S1: VIF values of full model

| Variables | GVIF | DF | GVIF^(1/(2*Df)) |
| --- | --- | --- | --- |
| Elevation_std | 1.436362 | 1 | 1.198483 |
| Slope_std | 1.314272 | 1 | 1.146417 |
| DWS_std | 1.199931 | 1 | 1.095414 |
| DS_std | 2.071085 | 1 | 1.439126 |
| DR_std | 1.838937 | 1 | 1.356074 |
| IVI_std | 1.246094 | 1 | 1.116286 |
| NDVI_std | 2.601889 | 1 | 1.613037 |
| Soil type | 1.818391 | 1 | 1.348477 |
| Aspect | 5.464903 | 7 | 1.128975 |
| Soil color | 1.690800 | 3 | 1.091 |

Table S2: VIF values of full model except soil color

| Variables | GVIF | DF | GVIF^(1/(2*Df)) |
| --- | --- | --- | --- |
| Elevation_std | 1.374815 | 1 | 1.172525 |
| Slope_std | 1.290460 | 1 | 1.13598 |
| DWS_std | 1.191727 | 1 | 1.091662 |
| DS_std | 1.979957 | 1 | 1.40711 |
| DR_std | 1.835645 | 1 | 1.35486 |
| IVI_std | 1.222764 | 1 | 1.10578 |
| NDVI_std | 2.566467 | 1 | 1.602020 |
| Soil type | 1.492102 | 1 | 1.221516 |
| Aspect | 4.395076 | 7 | 1.111543 |

**Table S3.** Summary statistics (mean ± standard deviation, SD) of environmental and habitat variables across all sampled grid cells (n = 101) and grid cells with pangolin burrows (n = 21) in Shivapuri Nagarjun National Park, Nepal.

| **Variables** | **Sampled grids (n=101)** | | **Burrow detected grid cells (n=21)** | |
| --- | --- | --- | --- | --- |
|  | **Mean** | **SD** | **Mean** | **SD** |
| Elevation (m) | 2073.03 | 180.26 | 2076.54 | 170.73 |
| Slope (°) | 19.70 | 5.74 | 19.73 | 5.87 |
| Nearest distance to water source (m) | 1329.25 | 1008.18 | 1393.28 | 1022.06 |
| Nearest distance to settlement (m) | 576.80 | 407.88 | 566.17 | 384.90 |
| Nearest distance to road (m) | 247.26 | 254.80 | 223.14 | 261.00 |
| Important Value Index (m) | 116.74 | 57.16 | 116.22 | 57.30 |
| NDVI | 0.28 | 0.09 | 0.29 | 0.09 |

Note: Elevation is expressed in meters above sea level (m a.s.l.), slope in degrees (°), and distances to water sources, settlements, and roads in meters (m). The Important Value Index (IVI) combines relative density, frequency, and dominance of tree species. The Normalized Difference Vegetation Index (NDVI) represents vegetation greenness, with higher values indicating denser vegetation.

**Table S4**. Poisson generalized linear model examining effects of environmental and habitat variables on active pangolin burrow counts across surveyed grid cells in Shivapuri Nagarjun National Park, Nepal.

|  | **Estimate** | **Std. Error** | **z value** | **Lower CI** | **Upper CI** | **Pr(>\|z\|)** |
| --- | --- | --- | --- | --- | --- | --- |
| (Intercept) | -3.3754 | 0.5133 | 6.493 | -4.3943 | -2.3566 | < 0.001 *** |
| DWS | 0.4887 | 0.2692 | 1.793 | -0.0454 | 1.0227 | 0.0729 |
| Elevation | -0.7157 | 0.3620 | 1.953 | -1.434 | 0.0026 | 0.0508 |
| NDVI | 1.4316 | 0.3757 | 3.763 | 0.686 | 2.1772 | <0.001*** |
| Slope | 0.9373 | 0.3588 | 2.588 | 0.2273 | 1.6472 | 0.0097 ** |
| DR | 0.4846 | 0.3985 | 1.200 | -0.3067 | 1.2758 | 0.2301 |
| IVI | 0.4178 | 0.3002 | 1.375 | -0.1779 | 1.01347 | 0.1693 |

Note: Estimates are model coefficients on the log scale, with standard errors (Std. Error), z values, and two-sided p-values [Pr(>|z|)]. Lower CI and Upper CI are the bounds of the 95% confidence intervals. Predictor variables include distance to water source (DWS), elevation (m), Normalized Difference Vegetation Index (NDVI), slope (°), distance to road (DR), and Important Value Index (IVI). Significance levels: *** p < 0.001, ** p < 0.01.

Figure S1: DHARMa nonparametric dispersion test via of residuals fitted vs. simulated of Poisson error distribution


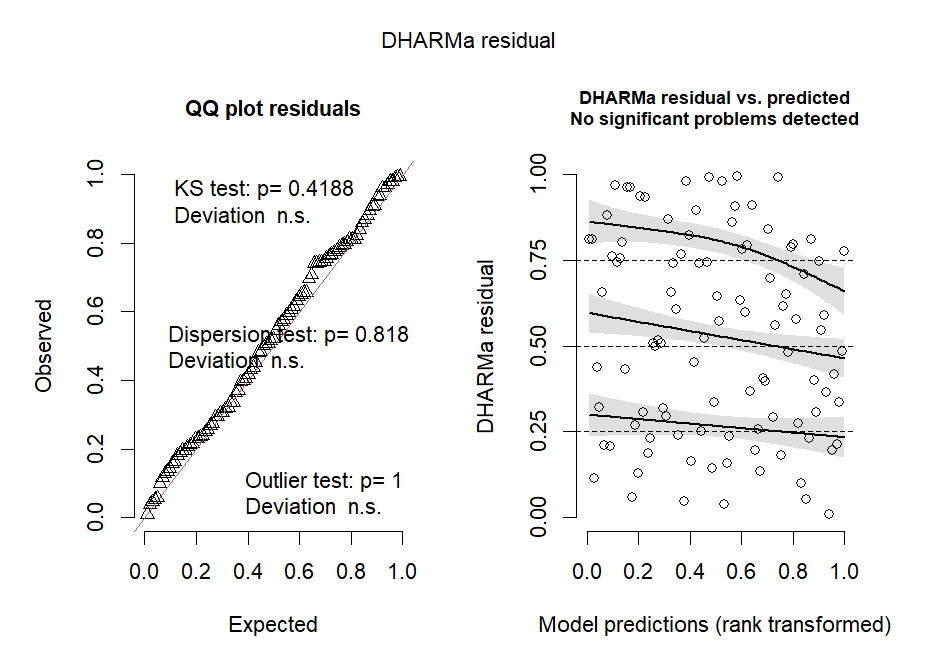


Figure S2: DHARMa zero-inflation test via comparison to expected zeros with simulation in Poisson error distribution


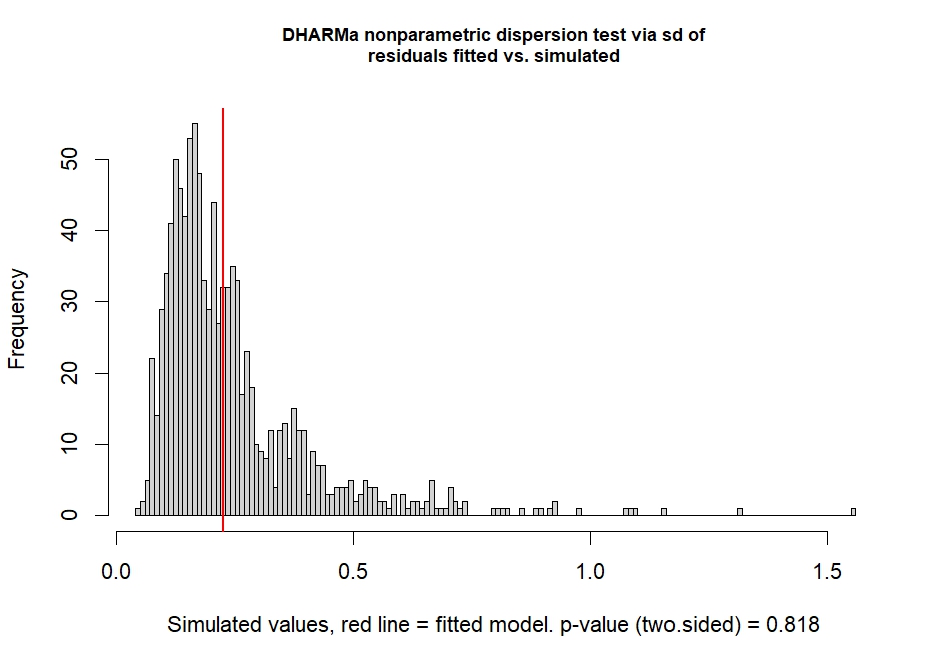


Figure S3: DHARMa nonparametric dispersion test via of residuals fitted vs. simulated of negative binomial GLM


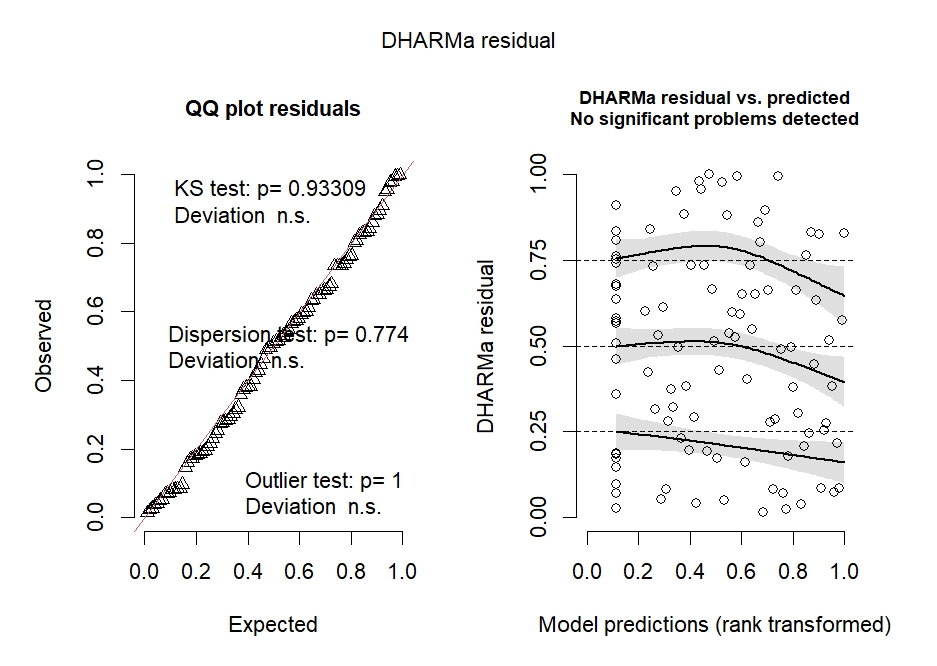


Figure S4: DHARMa zero-inflation test via comparison to expected zeros with simulation in negative binomial GLM


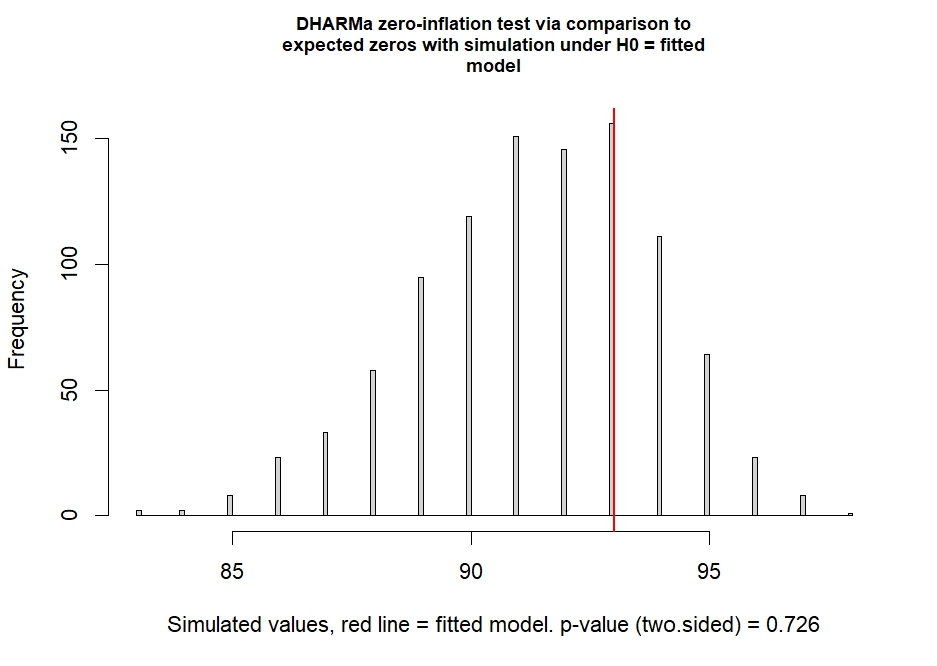


**Table S5.** Chinese pangolin burrows recorded under different ecological conditions

| Elevation (m) | 1750–1950 | 1950–2150 | 2150–2350 |  |  |  |  |  |
| --- | --- | --- | --- | --- | --- | --- | --- | --- |
| Number of burrows | 30 | 33 | 4 |  |  |  |  |  |
| Slope (°) | 0–15 | 15–30 | 30–45 |  |  |  |  |  |
| Number of burrows | 17 | 32 | 18 |  |  |  |  |  |
| Distance to settlement (m) | 0–700 | 700–1400 | 1400–2100 |  |  |  |  |  |
| Number of burrows | 56 | 10 | 1 |  |  |  |  |  |
| Distance to water body (m) | 0–700 | 700–1400 | 1400–2100 | 2100–2800 | 2800–3500 | 3500– 4200 |  |  |
| Number of burrows | 13 | 10 | 1 | 19 | 5 | 19 |  |  |
| Distance to road (m) | 0–200 | 200–400 | 400–600 | 600–800 |  |  |  |  |
| Number of burrows | 31 | 33 | 2 | 1 |  |  |  |  |
| Aspect | East | West | North | South | N-E | N-W | S-E | S-W |
| Number of burrows | 19 | 24 | 3 | 3 | 0 | 3 | 14 | 1 |
| Soil color | Brown | Black | White |  |  |  |  |  |
| Number of burrows | 61 | 5 | 1 |  |  |  |  |  |
| Soil type | Sandy | Loamy |  |  |  |  |  |  |
| Number of burrows | 37 | 30 |  |  |  |  |  |  |
